# Negative expectations interfere with the analgesic effect of safety cues on pain perception by priming the cortical representation of pain in the midcingulate cortex

**DOI:** 10.1371/journal.pone.0180006

**Published:** 2017-06-30

**Authors:** Abeer F. Almarzouki, Christopher A. Brown, Richard J. Brown, Matthew H. K. Leung, Anthony K. P. Jones

**Affiliations:** 1 Physiology Department, Faculty of Medicine, King Abdulaziz University, Jeddah, Saudi Arabia; 2 Department of Psychological Sciences, Institute of Psychology, Health and Society, University of Liverpool, Liverpool, United Kingdom; 3 School of Psychological Sciences, University of Manchester, Manchester, United Kingdom; 4 Royal Bolton NHS Foundation Trust, Farnworth, Bolton, United Kingdom; 5 Human Pain Research Group, Division of Neuroscience and Cognitive Psychology, University of Manchester, Salford Royal NHS Foundation Trust, Salford, United Kingdom; University of Reading, UNITED KINGDOM

## Abstract

It is well known that the efficacy of treatment effects, including those of placebos, is heavily dependent on positive expectations regarding treatment outcomes. For example, positive expectations about pain treatments are essential for pain reduction. Such positive expectations not only depend on the properties of the treatment itself, but also on the context in which the treatment is presented. However, it is not clear how the preceding threat of pain will bias positive expectancy effects. One hypothesis is that threatening contexts trigger fearful and catastrophic thinking, reducing the pain-relieving effects of positive expectancy. In this study, we investigated the disruptive influence of threatening contexts on positive expectancy effects while 41 healthy volunteers experienced laser-induced heat pain. A threatening context was induced using pain-threatening cues that preceded the induction of positive expectancies via subsequent pain-safety cues. We also utilised electroencephalography (EEG) to investigate potential neural mechanisms underlying these effects. Lastly, we used the Fear of Pain Questionnaire to address whether the disruptive effect of negative contexts on cued pain relief was related to the degree of fear of pain. As predicted, participants responded less to pain-safety cues (i.e., experienced more pain) when these were preceded by pain-threatening cues. In this threatening context, an enhancement of the N2 component of the laser-evoked potential was detected, which was more pronounced in fearful individuals. This effect was localised to the midcingulate cortex, an area thought to integrate negative affect with pain experience to enable adaptive behaviour in aversive situations. These results suggest that threatening contexts disrupt the effect of pain relief cues via an aversive priming mechanism that enhances neural responses in the early stages of sensory processing.

## Introduction

It is well established that the experience of pain is often influenced by expectations of its intensity [1] Many studies have identified that “positive” expectancies (e.g. expecting pain reduction) are associated with reduced levels of pain and improve treatment outcomes [2–5], whereas “negative” expectancies (e.g., expecting increased pain) have the opposite effect [6]. Similar phenomena are observed with placebo and nocebo manipulations respectively [7,8]. It is thought that such expectancy effects reflect a neural system that uses prior knowledge to make predictions about future sensory inputs, thereby enabling appropriate and timely behaviour [9–12].

Models of chronic pain such as the fear-avoidance model suggest that negative expectancy and fear are part of a maladaptive cognitive cycle that maintains chronic pain symptoms, suffering and disability [13]. In this model, fearful individuals who experience, observe or expect pain, may become hypervigilant in response to potentially painful stimuli and start to avoid activities that are expected to result in pain [13]. Further understanding the neurophysiological relationship between fear, negative expectancy and pain-associated behaviour may identify further targets for interventions in the management of chronic pain.

Studies have identified that negative expectations about pain outcomes disrupt the therapeutic effect of pain interventions (such as medications and physical therapies), seemingly negating any positive expectations delivered as part of the treatment [14, 15]. However, it is still unclear how the hypoalgesic effect of suggestions for reduced pain alone, are biased when there is a pre-existing expectation for increased pain. Aversive conditioning paradigms are often used to investigate the neural mechanisms of perceptual decision-making in the context of pain expectancy. As far as we are aware, no published study has used a conditioning model to investigate how pain-threatening cues influence positive expectancy effects at behavioural and neurophysiological levels.

In this study, we utilise electroencephalography (EEG) to investigate how prior negative expectancy cues influence the neurophysiological response to positive expectancy cues. By utilising the high temporal resolution of EEG we were able to investigate whether this effect influenced electrophysiological responses during sensory processing. Laser Evoked Potentials (LEPs) have been extensively used in the study of the neurophysiological effects of expectation and pain [16]. Evidence suggests that certain components of LEPs, which include the N2 and P2 peaks, are markers for the saliency of a stimulus, i.e. its ability to capture attention [17]. It is believed that highly salient stimuli are processed in a way that enables rapid and appropriate adaptation of an organism to its environment [17]. It has been identified that expectation, attention and emotion are able to influence LEP responses. For example positive expectancies generated by the delivery of a placebo cream have been demonstrated to decrease the P2 peak [18,19]. Moreover, theN2 peak, which is thought to be more related to the sensory aspect of pain [20, 21]; has also been shown to be modulated by cognitive factors, such as attention and expectancy [22].

On this basis, we firstly hypothesized that on a behavioural level, the hypoalgesic effect of positive expectancy cues on pain perception would be diminished when preceded by negative expectancy cues. Secondly, we hypothesized that this effect would be associated with a greater magnitude of EEG evoked potentials, reflecting a greater saliency of stimuli in a prior negative expectancy condition. Finally we hypothesized that individuals with the greatest fear of pain will demonstrate a more marked response to these conditions than individuals with a low fear-of pain.

## Methods

### Overview of procedure

The study consisted of a single session (lasting about 4 hours) where participants were informed about the nature of the laser-pain induced during the experiment, but were kept blind to its specific aims and objectives. Each participant began by providing informed written consent, before undergoing a psychophysics test to calibrate the pain intensities used in the main experiment for that individual. They then underwent a training procedure to set up the expectancy manipulation before the EEG cap was applied and they completed the main experiment.

### Participants

Forty-four participants (25 males, 19 females; mean age = 25.6 years, SD = 6.7, (aged 19–41 years old) completed the study. Most volunteers were recruited via paper and electronic adverts placed at the University of Manchester. Two of the volunteers reported being left-handed; the remaining 42 participants described themselves as being right-handed. The inclusion criteria stated that volunteers would be above 18 and pain free. The exclusion criteria included chronic pain, morbid psychiatric illness, neurological illness, ischemic heart disease, peripheral vascular disease, chronic skin disease (e.g., eczema, psoriasis) and hypertension not controlled by medication. After the experiment, one female volunteer reported she was taking pain killers for Irritable Bowel Syndrome and her data were excluded. Accordingly, complete data from 43 participants were available for analysis (although only 41 were included in the final analysis, due to 2 participants failing the manipulation check—see *Self Report Measures*). Each volunteer received ten pounds per hour, in addition to travel expenses, for their involvement. Ethical approval was given by the North West 9 Research Ethics Committee in the United Kingdom.

### Study design and procedure

**Fig 1:**
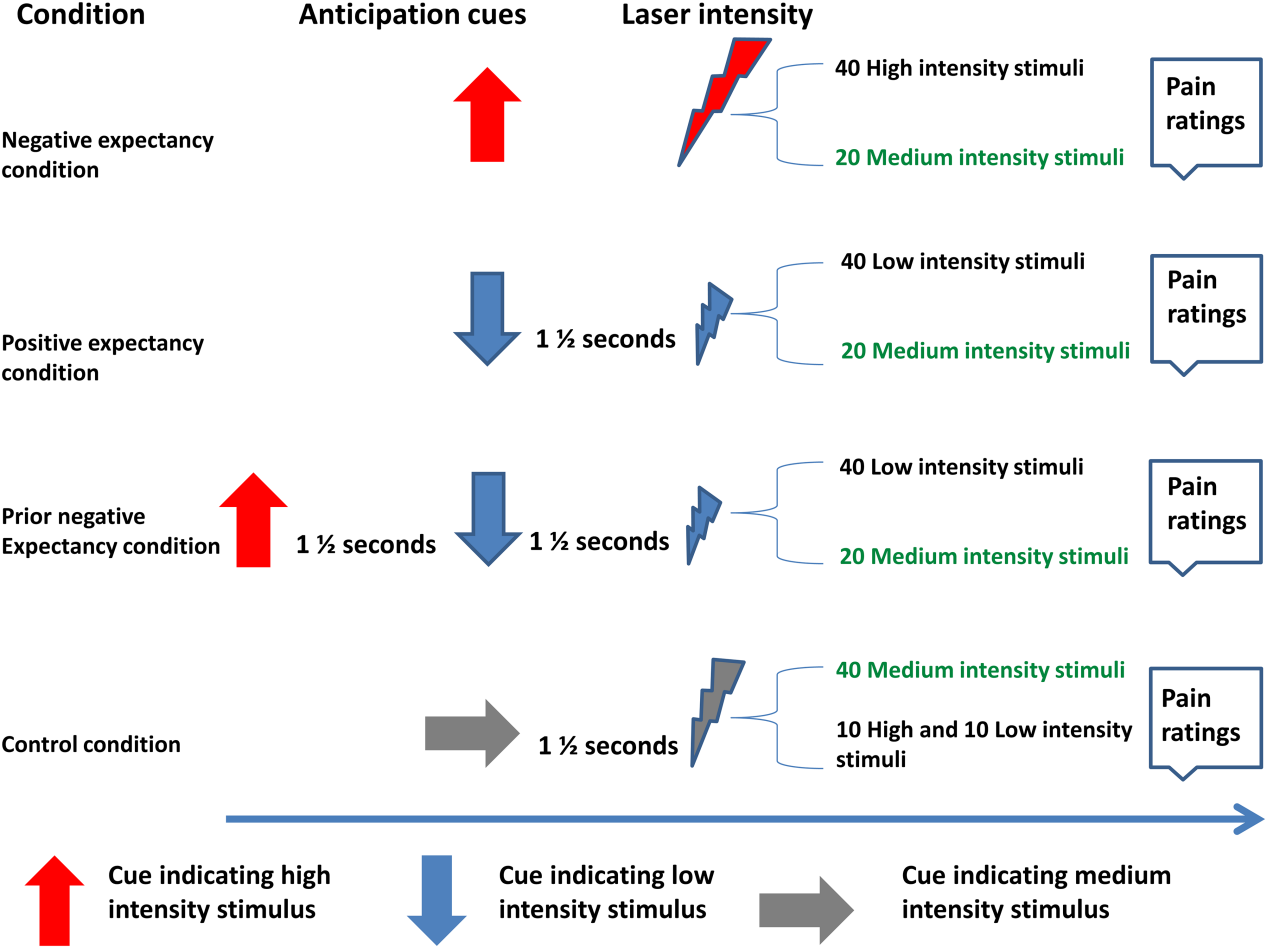
The expectancy paradigm. Participants were informed that the cue just preceding the pain was predictive of the upcoming pain. However, they were not informed about the occurrence or intensity of the ambiguous stimulus. All stimuli were followed by a numerical pain scale that was displayed for 6 seconds.

A within-subjects design was used. The experimental session consisted of 240 trials divided into four blocks of 60 trials each; these were interspersed with rest periods (lasting up to a maximum of 10 minutes) if participants wished (Fig 1). Rest periods were offered to all participants, however, were not incorporated into the experimental protocol, as many participants actively requested to continue, due to the long length of the experiment. Trials belonged to one of four expectancy conditions: positive expectancy, negative expectancy, prior negative expectancy and control expectancy. The prior negative expectancy condition was designed to reflect the clinical situation in which negative expectation may reduce the benefits of a subsequent treatment. In this study, the “treatment” was in fact a positive suggestion of the pain outcome, analogous to providing an inert placebo treatment. The control condition (for the prior negative expectancy condition) in this experiment was therefore a situation in which the positive “treatment” was provided but without the context of a pre-existing negative expectation (our “positive expectancy” condition). A condition such as a prior positive expectancy condition was considered, in which a positive cue might be followed by a negative cue. However, it was considered this may have undermined the trust or validity of the positive cue compromising the main aim of the study.

Trials corresponding to the different expectancy conditions were randomised over the course of the experiment. During each trial a computer monitor was used to display the anticipation cues. Participants were instructed to pay attention the screen the full length of the trial. Participant’s attention to these cues was monitored subsequently in the manipulation check (see section below). During positive expectancy trials, participants were presented with a single safety cue (downward arrow) that signalled an upcoming low-intensity stimulus. On negative expectancy trials, participants were presented with a single threatening cue (upward arrow) that signalled an upcoming high-intensity stimulus. This latter condition was included to reinforce participants’ expectations concerning the threat value of the upward arrow, ensuring a valid assessment of the prior negative expectancy effect. The negative expectancy condition was used to check the validity of the paradigm for manipulating expectations using behavioural data, but was not analysed inferentially for the purposes of understanding neural mechanisms. On prior negative expectancy trials, participants were presented with two cues: an initial threatening cue (upward arrow) that signalled an upcoming high-intensity stimulus, which was subsequently followed by a safety cue (downward arrow) indicating that a low-intensity stimulus would be presented instead. The aim of this condition was to experimentally simulate a real life scenario in which a person may have prior negative predictions regarding their pain outcome (i.e. high intensity pain), but then receive subsequent positive cues of relief from impending high pain that conflict with their predictions. Only this condition would then be compared with the positive expectancy condition, in order to test our hypothesis.

The control condition trials had a sideways arrow signalling a medium-intensity stimulus; this was included in order to evaluate whether the expectancy manipulations were having the desired effects in terms of pain-reporting behaviour. The primary outcomes were pain assessed by verbal report and Laser Evoked Potentials (LEPs).

In two-thirds of each expectancy condition trials, the cues were followed by a stimulus intensity that was congruent with the expectancy cue preceding it (i.e. the cue accurately predicted the stimulus intensity). In one-third of the trials, the cues were followed by a medium-intensity stimulus that was incongruent with the expectancy cue and the order of these was randomised within each trial. These were the critical trials enabling us to evaluate the effect of the expectancy cues on pain perception. For the purpose of EEG analysis, a minimum number of 20 critical trials are required to detect measurable LEP peaks (N2 and P2) [23]. Keeping in mind the maximum number of pulses that could be given without harming volunteers, a ratio of 40:20 (cue-consistent intensity trials: medium intensity trials) pulses was used. Only the 20 medium-intensity trials were analysed. A different ratio was used for the control condition to ensure equal numbers of low- and high-intensity stimuli (see Fig 1); here two-thirds of the trials used medium-intensity stimuli (as predicted by the cue) and the remaining third was a mix of low and high-intensity stimuli.

Each trial started with an expectancy cue presented on a computer monitor, 2 seconds after which the subject received a laser pulse to the back of the right arm which was at one of three possible individually-calibrated intensity levels: high, medium, or low. For the prior negative expectancy condition only, there were two expectancy cues and a laser pulse was administered 2 seconds after the second cue (Fig 1). Participants were asked to rate their pain using a scale presented on the monitor 3 seconds after the laser pulse which was displayed for 6 seconds. Volunteers used a button pad to report their pain response.

### Psychophysics test

In order to individually calibrate the stimulus intensity levels required for the experiment, participants performed a psychophysics test before the experiment proper. In this test, the pain scale was explained to participants and participants then received consecutive laser pulses, which they rated according to the scale. Each participant was given ascending intermittent levels of laser stimuli starting from imperceptible to a maximum pain tolerance as decided by the volunteer. In this way, three levels of pain were determined for use in the experiment: Level 3 (low-intensity stimulus), which was described as hot but not painful; Level 5 (medium-intensity stimulus), which was described as low, ignorable pain; and Level 7 (high-intensity stimulus), which was the maximum level of pain volunteers were prepared to experience during the experiment. The psychophysics protocol as well as the selected stimulus levels followed identical protocols of previously published laser-pain experiments [24, 25, 26]. There was no pairing of the laser stimulus with cue at this stage as the aim of the test was only to individually calibrate laser energy to account for individual variations in pain threshold and tolerance regardless of the associated cue.

### Laser stimuli

The stimuli were delivered to the right forearm using a thulium laser which had a beam diameter of 6 mm, and a pulse duration of 100 milliseconds. The stimuli were randomly moved around this area to avoid skin damage, habituation and sensitisation as described previously in similar experiments [24,25]. Participants wore protective safety goggles at all times. Further tuning of the pain stimulus to ensure consistency was performed as described elsewhere [27]. Laser energy output (mean (standard deviation)) across participants for level 7 was 1.1 (0.2) Joules, for a level 5 was 0.7 (0.1) Joules, and for a level 3 was 0.3 (0.07) Joules.

### The training procedure

In order to enhance the validity of the expectancy cues, participants completed a training procedure after the psychophysics test and prior to the experiment proper. During the training procedure, volunteers were shown the expectancy cues, told what they predicted and subsequently presented with laser stimuli that were matched with their associated intensity. For example, to create a positive expectancy effect, participants were presented with a downward arrow, were told that this cue predicted a subsequent low-intensity stimulus, and were presented with a low-intensity laser stimulus. We did not train subjects on the “medium” stimuli as this condition was designed to be deliberately ambiguous, so that participants susceptible to expectancy effects would be more likely to rely on prior expectations. There were fifteen trials of this training procedure, 5 for each cue-stimulus combination. Participants were informed that for each trial in the experiment proper, the trial would start with an initial arrow that would inform them about the intensity of the subsequent stimulus. They were also informed that the arrows would not normally change, but that if they did it would be the second arrow, not the initial arrow, that predicted the intensity of the upcoming stimulus. Participants, on average, rated a level 3 pain stimuli as 3.3 level 5 as 4.8 ± 1.2 and finally a level 7 stimuli as 5.8 ± 0.9. Participants confirmed verbally that they understood the procedure before continuing with the main experiment.

### Self-report measures

#### Pain scale

**Fig 2:**
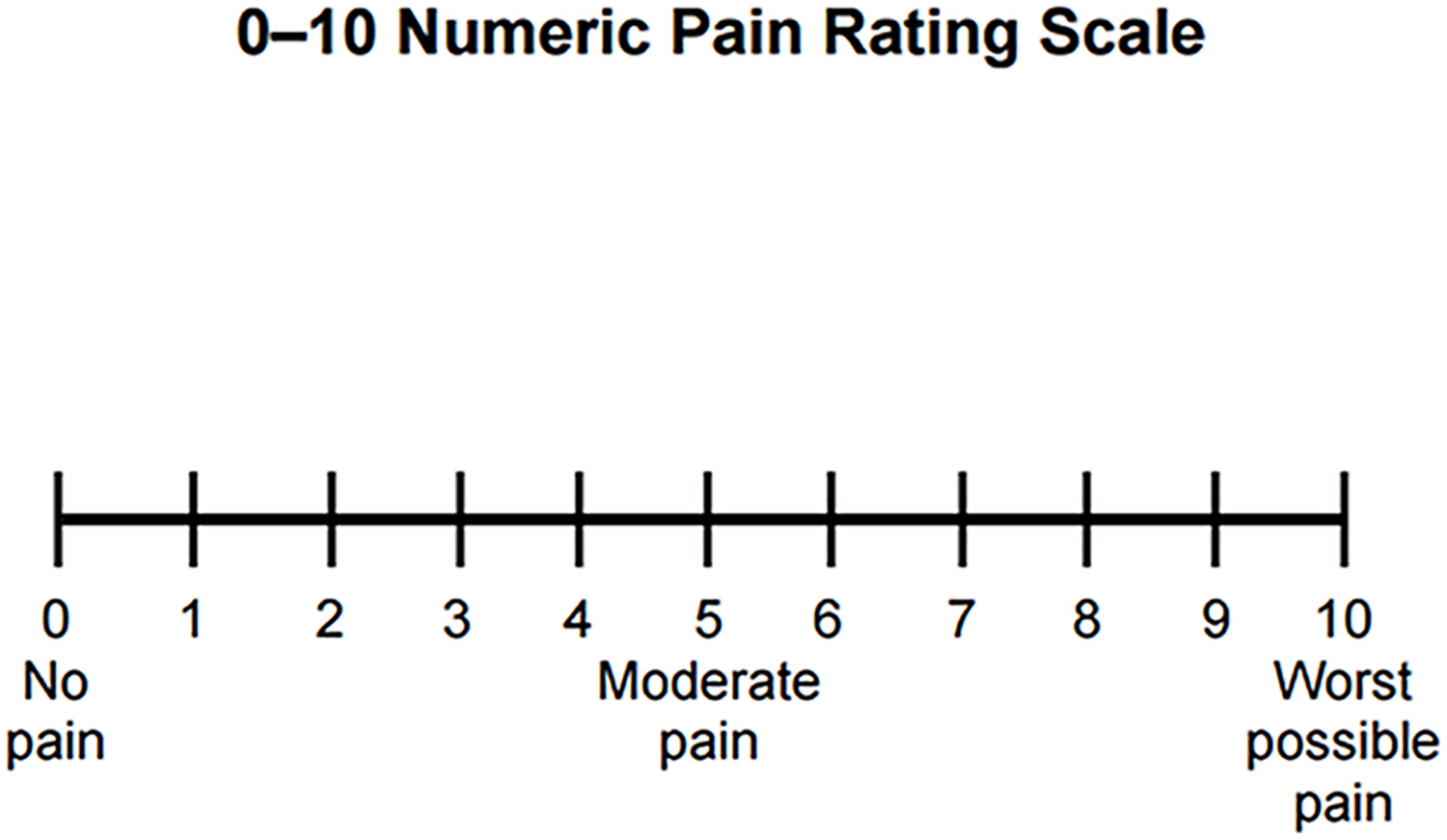
The pain scale.

The main behavioural outcome measure was an 11-point (0–10) numerical pain scale (Fig 2). Participants were asked to rate the stimulus on a 0–10 pain numerical scale, where 0 indicated “no pain”, 10 indicated “extreme pain” and 4 was the pain threshold. In order to decrease ambiguity and to ensure a consistent approach to rating, each number in the scale was explained in relation to a sensation from everyday life. The number 3 on the scale (which signifies a low-intensity; non-painful stimulus) was explained to volunteers as a very hot, but not a painful, sensation; number 4 was the intensity in which the laser stimuli first started to feel painful, as opposed to very hot; number 5 on the scale (medium-intensity stimulus) was explained as a low, ignorable pain; finally, number 7 (high-intensity stimulus) was explained as the maximum pain volunteers would want to experience during the study and would not be happy to go beyond this. This pain scale and pain level descriptions were in line previously published experiments using the same numerical pain scale [24, 25,27].

#### Fear of Pain Questionnaire-III (FPQ-III)

Pain-predictive cues trigger fear, to allow the individual to avoid the harm associated with the cue predicting it. The fear-avoidance model of chronic pain is a model that describes the relationship between fear, negative expectancy and pain [13]. According to this model, when pain is interpreted as evidence of bodily harm or danger, it demands attention and interrupts ongoing activities [28]. These factors initiate avoidance behaviour, that over time, contributes to aversive conditioning and reinforces further pain experiences, negative expectancies and avoidance [13]. It is therefore crucial to assess for fear, if we are to better understand the mechanisms by which negative expectancies influence pain outcome. In the context of this study, the fear of pain questionnaire was specifically selected, to assess whether any expectancy effects were associated with fear of pain [29]. This 30-item questionnaire measures participant’s fear of pain across three domains; minor, severe and medical pain, using a 5-point Likert response scale, ranging from zero “not at all”, to five “extremely”. The questionnaire consists of separate subscales for fear of minor pain, fear of medical pain and fear of severe pain, which can be calculated separately or combined to form a total score [29]. The validity and internal consistency have been tested and confirmed previously [30]. Participants completed the questionnaire before performing the main experiment but after the psychophysics test.

#### Manipulation check

The cornerstone of this study design is the expectancy cues; the validity of any results is based on the integrity of these cues. It was essential to make sure that volunteers were responding to the cues adequately and properly. This was addressed by giving volunteers a three-item, expectancy manipulation check measure at the end of the experiment, designed to assess the following: attention directed to the cues, reliability of the cues as predictors of the upcoming stimulus and subjective report bias by the cues. The questionnaire consisted of three questions: (i) During the task, how much did you focus on the direction of the arrows? (ii) How accurately did the arrow cues predict the intensity of the pain that followed? (iii) When rating the intensity of the pain, how much was your rating based on the direction of the preceding arrow cue? In each case, participants were asked to select an answer from a scale starting with “not at all” to “all the time”. Participants, who reported ignoring the cues completely, or most of the time (>75% of the trials were ignored), were excluded from the analysis. Two participants were excluded from the analysis for this reason, leaving a total of 41 participants for further data analysis. Although it would have been more accurate to measure cue manipulation trial-by-trial, this would have been practically infeasible (by considerably extending the length of the experiment) and may have distracted participants’ attention from the expectancy cues.

### Electroencephalographic recordings of laser evoked potentials

Laser Evoked Potentials (LEPs) were measured with EEG. EEG recordings were obtained from 59 Ag/AgCl surface electrodes attached to an elastic cap placed in accordance with the extended international 10–20 system (Brain Vision Acticap combined with a Neuroscan head box and amplifier system). Band-pass filters were set at DC—100Hz, with a sampling rate of 500Hz and gain of 500. A notch filter was set to 50Hz to reduce electrical interference. Electrodes were referenced to the ipsilateral (right) earlobe. The horizontal and vertical electro-oculograms (EOG) were measured for detection of eye-movement and blink artefacts.

## Data analysis

### LEP analysis

EEG data were analysed using Brain Vision Analyzer 2.0. Most of the steps followed for the analysis were in accordance with previously published work [25, 27, 31, 32]. Data was first down-sampled to 125 Hz/second. The time of laser stimulation served as 0ms. Data were segmented 500 milliseconds before the cue predicting the pain (the second cue in the prior negative expectancy condition) and 1500 ms after the laser stimulus, i.e. the length of each epoch was two seconds. Linear trends were removed from the data by using the first and last 500ms of the epoch. Blink and horizontal eye movement artefacts were removed using Independent Components Analysis. Filtering was then applied to each epoch at a high cut-off of 30Hz and 12OCT/D. Data were averaged across all participants for each condition separately (medium-intensity stimuli only). Epochs were re-referenced to the common average (i.e., the average voltage over all electrodes). N2-P2 LEP component peaks were detected for each participant in reference to electrode Cz where the N2 and P2 peak topographies were identified to be maximal. The N2 peak amplitude was specified as the largest negative deflection in a defined time period between 200ms and 350 ms after laser stimulation, while P2 amplitude was the largest positive deflection between 350ms and 500ms. Although N1 may have served as a better marker for early cortical responses [33], it was not elicited in our data in enough subjects to be analysed. This is likely because we used a relatively long pulse duration for the laser (100ms) by the standards of other LEP researchers who may go down to 20ms or so. We use 100 ms pulse duration based on our observations that longer stimulus durations results in less skin damage. Long stimulus durations are likely to blur the N1 peak over time so that it is not distinct enough from the background noise. Data were pooled from the nine central electrodes (CPz, Cz, FCz, CP1, CP2, C1, C2, FC2, FC1) and a grand average was produced from the analysed trials for LEPs. Data from six participants were corrupted during saving and was not possible to analyse. One participant did not have an identifiable N2 deflection within the determined time period and was excluded from further peak analysis and the grand LEP average displayed. Data from two participants had artefacts that were not cleared after analysis, making it difficult to extract meaningful ERP information; these were also excluded from further analysis. Accordingly 32 participants’ data were used for LEP analysis.

### Source localisation analysis

Cortical sources of the LEP were estimated with low-resolution electromagnetic tomography (LORETA), using the LORETA-KEY software [34]. This was carried out on averaged data for each participant and each condition for LEP epochs. LORETA calculates the spatially smoothest source estimates compatible with observed EEG activity across all electrodes on the scalp. The EEG activity is mapped onto 2394 voxels in three-dimensional space. Each voxel represents a possible activity source. The comparison was made between the prior negative expectancy condition and the positive expectancy condition.

### Statistical analysis

Statistical analysis was performed using SPSS version 20. The results of this study are expressed as means (M) and standard deviations (SD) unless otherwise indicated. An alpha value of *p* < 0.05 (two-tailed) was considered significant. Histograms of the pain ratings at the four expectancy conditions showed an approximate normal distribution. The N2 peaks showed a non-normally distributed pattern, confirmed by significant results from a Shapiro-Wilk test, so the Wilcoxon Signed-Rank Test was used to compare means of N2 data. For LORETA results inferential statistics were done using non-parametric tests. The mean and standard deviations for pain ratings were obtained only from those trials with a medium-intensity stimulus. For paired tests, Cohen’s d effect size is reported; this value is considered small if 0.2, medium if 0.5 and large if 0.8. For LEP analysis, N2/P2 amplitudes values for each participant (mean values across 10ms either side of the peak; i.e. 20ms in total) for the medium-intensity critical trials were exported to SPSS.

#### Behavioural data inferential analysis

Initially, a repeated measures analysis of variance (ANOVA) was used to examine the effectiveness of the conditioning paradigm in generating expectancy effects on pain reports. The main factor of the ANOVA was expectancy which had four levels: control, negative, prior negative and positive expectancy. In the event of a significant main effect, post-hoc paired t-tests were performed comparing negative expectancy and positive expectancy conditions with the control condition (pain reports only). This was done to ensure the conditioning paradigm was successful and that individual expectancy cues had significantly induced expectancy effects on pain reports. Specifically, this included determining whether the cues manipulated the intensity rating for the negative and the positive expectancy condition (higher and lower pain rating, respectively) as previously found in other similar paradigms [32]. The main hypothesis was tested using a planned comparisons paired t-test comparing the mean pain ratings in the prior negative expectancy and positive expectancy conditions. Five planned t-tests were conducted on the behavioural results, therefore the threshold of significance was lowered for these tests to *p* = 0.05 / 5 = 0.01.

#### EEG data analysis

EEG is not a pain marker per se but represents a means of understanding the neurophysiological dynamics of nociception, including cognitive influences on nociception, and so served as a tool to investigate the neural mechanism of the effect of the prior negative expectancy on signals of pain relief (positive expectancy cues). On this basis, our aim in the EEG analysis was to selectively examine the effect of the prior negative cues on the positive safety cues at a neurophysiological level. The two conditions of interest were therefore the prior negative condition and the positive expectancy condition (EEG data from the negative expectancy condition was not intended for analysis). Statistics were performed using paired t-tests comparing prior negative expectancy and positive expectancy medium-intensity trials for N2 and P2 amplitudes separately. For LORETA analysis, to identify sources of the prior negative expectancy effect, sources of the two conditions of interest were compared by voxel-wise non-parametric test with correction for multiple comparisons, using a randomisation and permutation test as implemented in the LORETA-KEY software. This was again done for each peak separately.

For correlation of the expectancy-related change in LEP amplitude with FPQ scores, for each of the N2 and P2 peaks, LEP amplitudes from the prior negative expectancy condition were first subtracted from the positive expectancy condition.

## Results

### Pain reports

**Fig 3:**
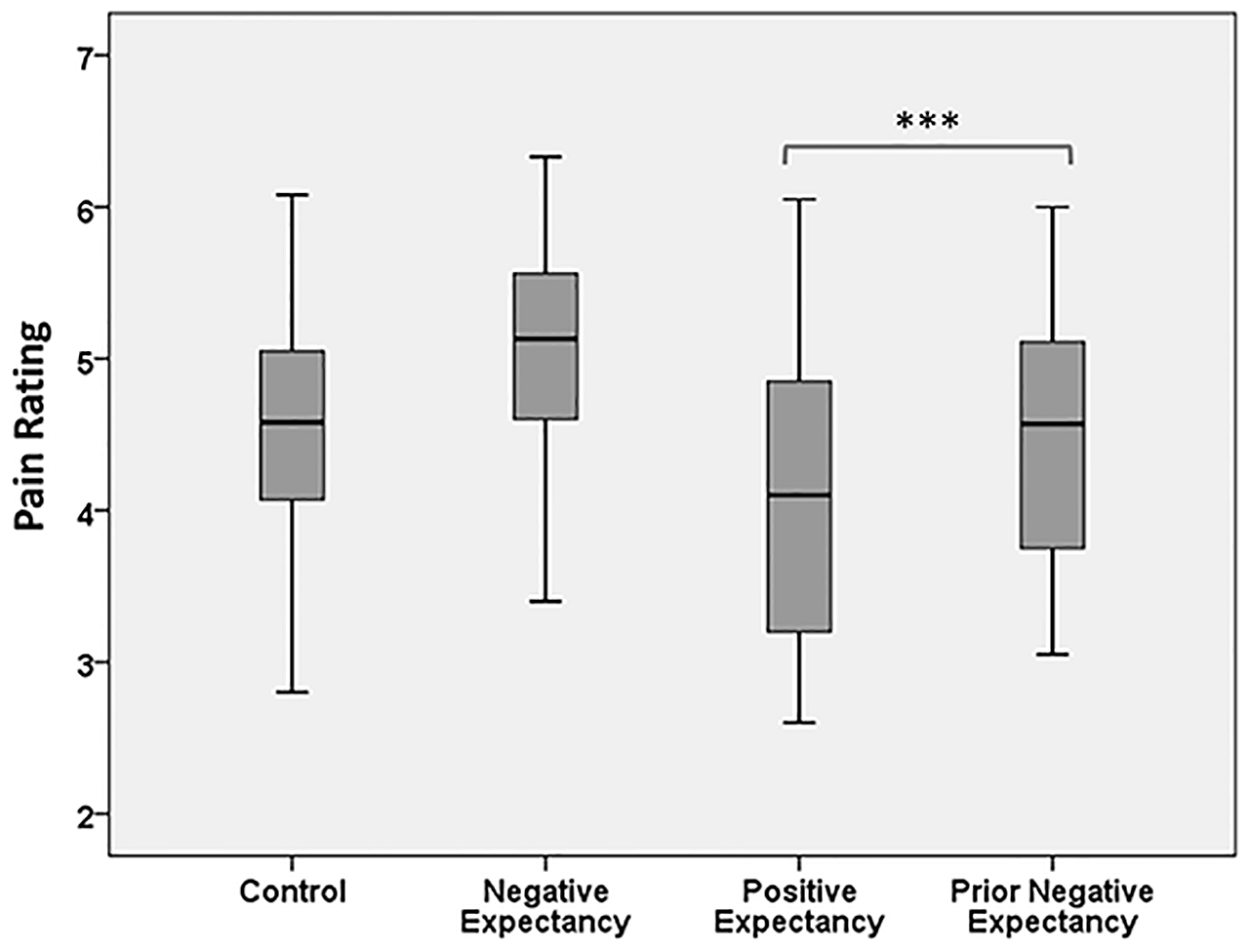
Pain ratings. Pain ratings for medium-intensity stimuli were the highest in the negative expectancy condition and lowest in the positive expectancy condition. Pain ratings in the prior expectancy condition were slightly lower than the control condition. *** p<0.001.

The behavioural results show that, as predicted, expectations significantly changed pain ratings. Expectations of high-intensity stimuli increased pain ratings of moderate-intensity stimuli, while expectations of low-intensity (non-painful) stimuli decreased pain ratings of moderate-intensity stimuli (Fig 3). ANOVA results showed a significant effect of expectancy cues [*F* (3, 40) = 24.41; *p* = 0.001, ηp^2^ = 0.977]. Planned paired t-tests revealed a significant difference between pain intensity ratings between the positive and prior negative expectancy conditions [*t* (40) = -4.54, *p* = 0.001, Cohen’s d = 0.71], such that pain was rated as higher when the positive cue was presented with a negative cue beforehand. Post-hoc t-tests for comparisons of the remaining conditions are displayed in Table 1 (corrected for multiple comparisons). Consistent with previous research [32], pain ratings were lower overall during the main experiment than the pain levels determined during the psychophysics test. This is has been observed in similar studies in which it was discussed that this may be due to habituation or a reduction in anxiety [32]. See S1 Dataset for individual pain reports scores.

**Table 1:** Post hoc comparisons for the different expectancy conditions.

Comparison	T value	P value	Effect Size (Cohen’s d)
Positive vs. Prior negative expectancy condition	-4.54	<0.001	0.71
Control vs. negative expectancy condition	-6.09	<0.001	0.95
Control vs. positive expectancy condition	3.57	0.001	0.55
Control vs. prior negative expectancy condition	0.73	0.466	0.11
Negative vs. positive expectancy condition	6.72	<0.001	1.04
Negative vs. prior negative expectancy condition	4.43	<0.001	0.69

### EEG results

#### Laser Evoked Potential (LEP)

**Fig 4:**
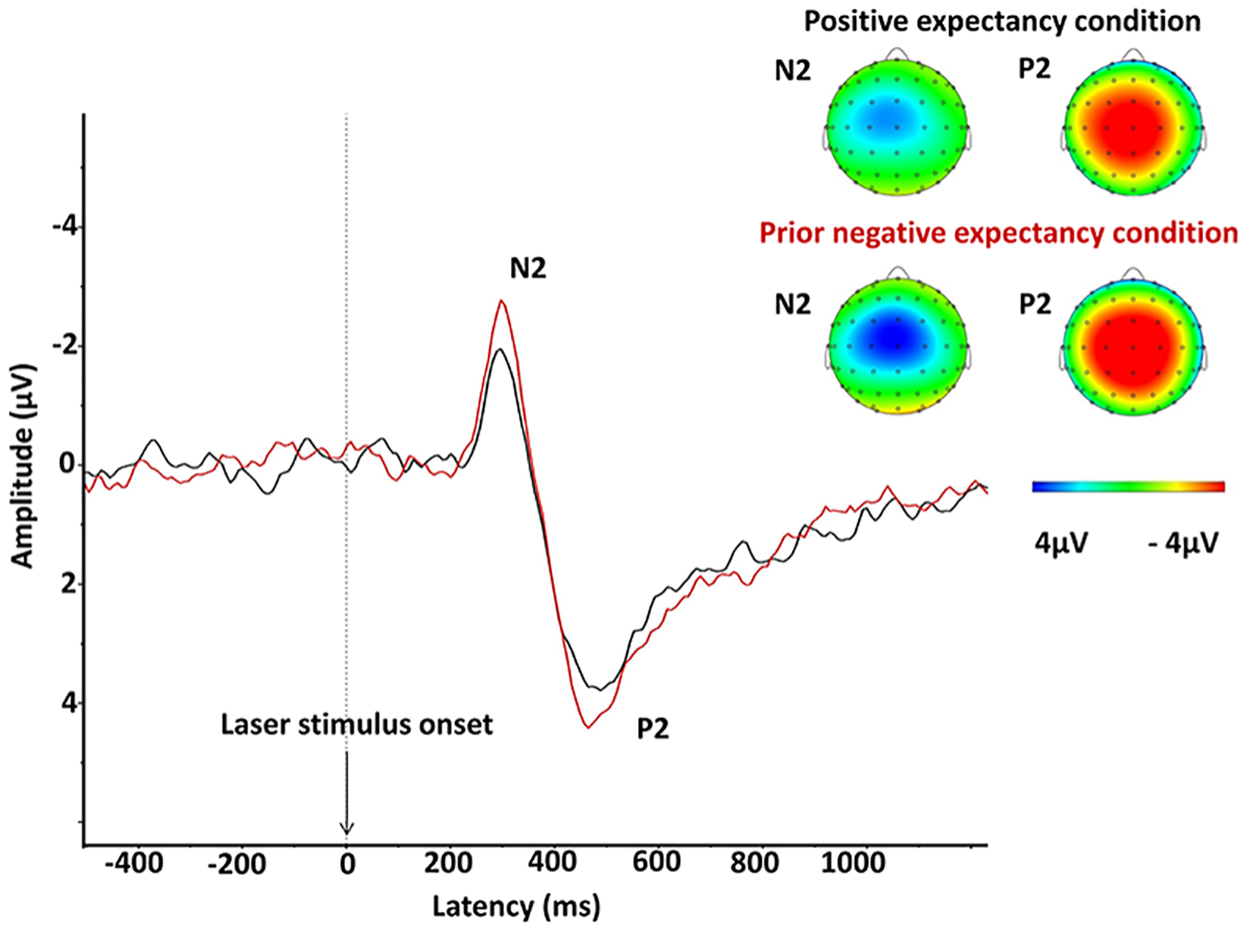
N2 magnitude differences between positive and prior negative expectancy conditions. Averaged LEP peak amplitude across all participants are displayed. Nine electrodes (FC1, FCz, FC2, C1, Cz, C2, CP1, CPz, CP2) were pooled around the central Cz to produce an average for each of the N2 and P2 peak potentials. The LEP in the positive expectancy condition is displayed in black. The LEP in the prior negative expectancy condition is displayed in red.

LEP data analysis showed a late, high-amplitude, negative-positive complex (N2/P2). The most dominant negative peak (N2) peaked around 288ms for the positive expectancy condition and 296ms for the prior negative expectancy condition (grand average); both peaks were maximal at electrode Cz. The most prominent positive peak (P2) peaked at approximately 464ms for both conditions and was maximal at Cz (Fig 4). N2 in the prior negative expectancy condition was -3.8 and the positive expectancy condition -2.5. Using a Wilcoxon Signed Rank Test, N2 amplitude was significantly larger for the prior negative expectancy condition compared to the positive expectancy condition [*Z* = -2.89, *p* = .004, effect size (*Z/√N*) = 0.43]. Expectancy modulation of P2 exhibited a similar trend, but was not significantly different between the two conditions. See S1 Dataset for individual laser evoked potentials amplitudes.

#### Source localisation for LEP (LORETA)

**Fig 5:**
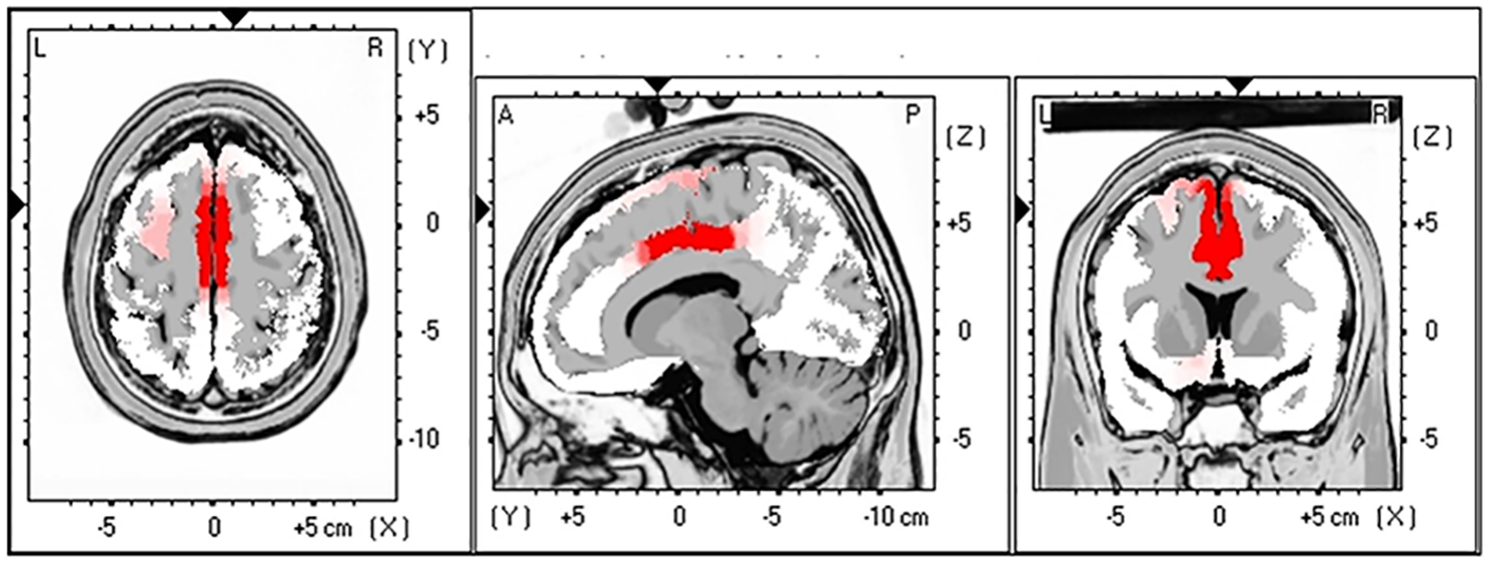
LORETA results. Source analysis contrasts following a paired t-test of N2 peak between the prior negative expectancy condition and the positive expectancy condition revealed activation of MCC at corrected significance threshold of p < 0.05.

Source localisation identified the midcingulate cortex (MCC) as the source of the neural activity contributing to the measured difference in N2 between the two conditions (Fig 5). This MCC activation correlated with the prior negative expectancy effect on N2 changes, *r* (31) = 0.45; *p* = 0.01, with larger changes in N2 amplitude being associated with stronger activation of MCC. No significant brain activation was evident for the contrasts made for P2.

### Fear analyses

**Fig 6:**
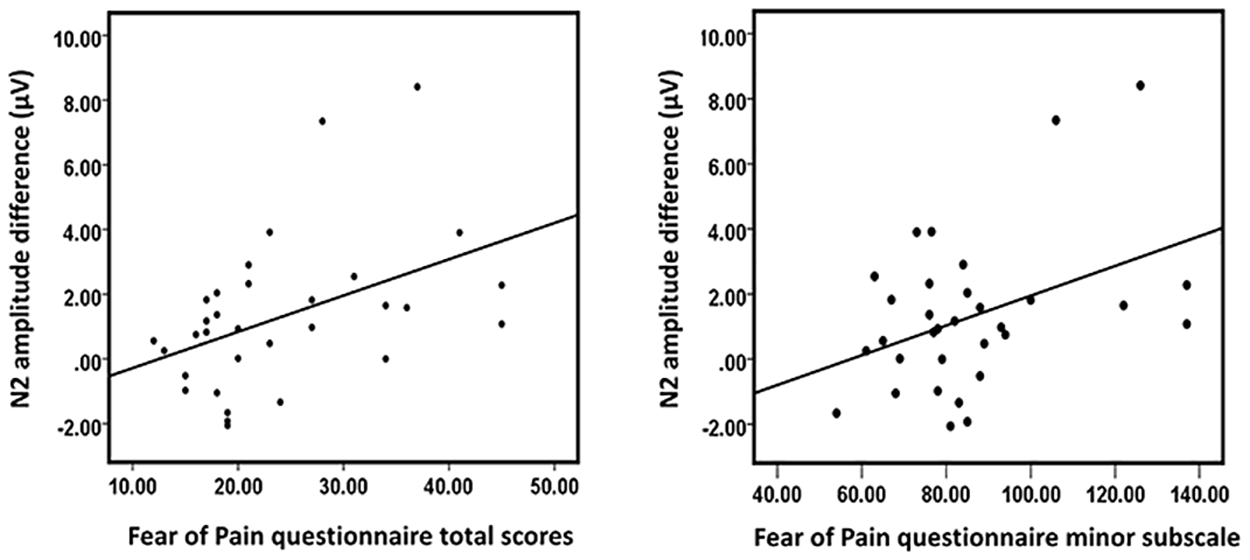
Scatterplot and linear least-squares line showing the correlation between the prior negative expectancy effect on N2 amplitude (prior negative expectancy condition minus positive expectancy condition) and the scores of the Fear of Pain Questionnaire (FPQ).

The prior negative expectancy effect on N2 (the difference in N2 amplitude between the prior negative and positive expectancy conditions) was significantly positively correlated with total scores on the fear of pain questionnaire (FPQ), *r* (32) = 0.40; *p* = 0.02, and the minor fear subscale *r* (32) = 0.44; *p* = 0.01 (Fig 6). See S1 Dataset for individual scores.

### Manipulation check

Two participants reported ignoring the arrows and were excluded from the analysis as mentioned above. Two participants left the scale unscored. In relation to the reliability of the cues, only two participants reported that the cues bore no relationship to the pain. As for report bias, none of the participants reported that their ratings were based on the direction of the arrow cues all or most of the time.

## Discussion

The main purpose of this study was to investigate whether, and how, prior threat cues (i.e. negative expectancies) disrupt the pain reducing effect of safety cues (i.e. positive expectancies) on pain perception. The first step was to validate the effect of positive (safety) and negative (threat) cues on medium intensity stimuli using a neutral control cue, which confirmed this effect. We then demonstrated that participants perceived laser stimuli as more painful when safety cues were preceded by threat cues, in comparison to when safety cues were presented alone. In other words, suggestions of pain reduction were less effective when participants had prior negative expectations regarding outcomes (i.e. expected the outcome to be more painful). The prior negative expectancy effect on pain ratings occurred alongside an enhancement of sensory processing, as demonstrated by increased amplitude of the N2 component of the LEP. This effect was most pronounced in individuals with the highest fear of pain and corresponded to enhanced activity in the MCC.

The distorting effect of prior negative expectations occurred despite participants being informed and conditioned to believe that subsequent safety cues were accurate in predicting the outcome. The findings are consistent with clinical and experimental observations describing the distorting effect of negative expectations on treatment outcomes including pain reduction [14, 15, 35]. This study, however, has the advantage of being able to establish a cognitive expectancy effect on a purely cognitive intervention in a controlled experimental setting. This allowed us to avoid the potentially confounding effects of pharmacological or physical interventions.

Previous studies have established that cues about incoming pain modulate pain sensation through adaptive pain modulatory networks, enabling appropriate adaptive behavioural responses [10]. In an aversive context, these mechanisms integrate threatening predictions with upcoming stimuli to increase the level of pain perceived, whilst in potentially safe contexts, predictions of less painful outcomes activate hypoalgesic mechanisms decreasing the level of pain perceived [10]. Such effects occurred in our study when presentation of a single safety cue decreased pain ratings of moderate-intensity stimuli, whilst presentation of a single threatening cue increased pain ratings of moderate-intensity stimuli. However, when a threatening cue preceded a safety cue, the pain rating was similar to the control (neutral) condition, suggesting that the hyperalgesic effect of the former interfered with the hypoalgesic effect of the latter, resulting in a decreased effect of the safety cues.

The neurophysiological results of the study indicate that threatening cues preceding safety cues augment early neural responses to thermal stimuli, as the amplitude of the N2 component of the LEP was increased. LEPs reflect the earliest cortical responses to thermal stimuli and have previously been shown to be modulated by cue-based expectancy [32]. The exact significance of LEPs is still debated [36], however motivational models of pain processing suggest that LEPs reflect the saliency (ability to capture attention) of nociceptive stimuli, which is dependent on both the stimulus characteristics and the motivational state of the individual [17]. Such models explain how LEPs are modulated by attention, expectancy and emotion, which will influence the initial early cortical representation and thereby subsequent evaluation of the pain stimuli, in relation to the current relevant motivational state [17]. That said, our results of an enhanced N2 suggest that in the condition of preceding threat, aversive pain modulatory networks are primed, altering the initial early cortical sensory representation of the thermal stimuli and increasing its saliency. Such priming effects have also been seen in the emotional modulation of LEPs, in which negative emotional cues prime individuals, leading to an enhancement of the early negative components of the LEP [37]. Furthermore it is important to note that this priming effect and enhancement of early sensory processing occurs despite subsequent safety cues.

The correlation between trait fear of pain and the prior threat effect on N2 suggests that individuals with high trait fear of pain have an increased propensity to activate mechanisms that enhance early nociceptive saliency in this condition. This is consistent with previous studies showing that individuals with high pain-related fear demonstrate an increased hypervigilance to threat related cues [38]. Previous work has also shown that healthy participants who are more afraid of pain pay more attention to negative pain messages than those who are less fearful [39, 40]. It is therefore possible that the most fearful individuals in the current study responded less well to the positive cue in the prior negative expectancy condition because they were more attentive to pain-threat signals [39]. The enhanced sensory processing observed here in fearful individuals may suggest that priming of aversive mechanisms occurs more readily in such individuals. Accordingly, these results may provide experimental evidence that supports and provides further insight into the fear-avoidance model of chronic pain [41]. This may also suggest that pain-related fear and negative expectations (i.e. catastrophic thoughts) regarding painful events contribute to the exacerbation of pain suffering over time, as well as avoidant behaviour and subsequent disability [13]. The fact that trait fear of pain was not correlated with subsequent pain ratings suggests that the increased sensitivity of salience networks to threatening contextual influences may not influence pain intensity directly, but may do so via subsequent maladaptive behaviour, as in the fear-avoidance model. These interpretations around the fear avoidance model however, need to be further investigated in a clinical setting in a patient population with chronic pain, as our current findings are confined to healthy subjects with a range of trait for fear.

The source localisation analysis identified that the effect of prior threat on the N2 was localised to the MCC. The MCC has been found to be associated with a number of different processes including, but not limited to, the anticipation and processing of pain, negative affect, cognitive control processes (such as prediction error monitoring), preparatory motor activity and attention [42]. A recent integrative model of the anterior MCC suggests it is an area of functional convergence of these processes, essential for the integration of information regarding punishment (i.e., pain and negative affect) into aversively motivated actions, in order to adaptively respond to threatening and uncertain situations [42]. Coordination of such actions occurs through integrating information regarding cognitive control, as well as strong connections with motor centres in order to plan and refine aversively motivated behaviours [42]. This “adaptive control” hypothesis of the MCC is consistent with our results, as it suggests that aversive predictions bias the pain reduction afforded by safety cues through mechanisms that alter the early cortical representation of sensory stimuli, in the MCC, in order to adaptively respond to the preceding threat or pain. Such adaptive responses to acute pain may be maladaptive for chronic pain, pointing to MCC activation as a potential target for therapeutic intervention, for example by neurofeedback [43]. In summary, the findings in this study provide important neurophysiological insights into negative preconceptions which may interfere with positive information about treatments, resulting in a reduced therapeutic outcome. Moreover, the enhanced priming of aversive mechanisms in fearful individuals identified in our study further strengthens the theory that pain-related fear may be an important target when managing pain.

## Limitations of the study

Previous work by Brown et al. found that certainty about forthcoming stimuli influences participants’ perception of the direction of the anticipatory cues [32]. MCC activation is reported in uncertain pain contexts [44]. Accordingly, it may be argued that there is more outcome uncertainty in the prior negative expectancy condition (due to the change of the cue) as compared to the positive expectancy condition, and that certainty in the positive expectancy condition favoured a lower stimulus rating as compared to the prior negative expectancy condition. Precision of predictions is thought to be an essential determinant of the expectancy modulation of pain, according to predictive coding models of pain processing [11]. The resultant effect of reduced certainty in cue-based predictions is a pain experience that is determined more by the bottom-up characteristics of the stimulus (intensity of stimulus) [32]. Such a mechanism would be consistent with our results, as the prior threat condition (contrasting cue condition) produced pain reports that were similar to the control condition. However, it is important to note that participants were informed that the second cue would predict the stimulus and a retrospective questionnaire after the experiment suggested participants on the whole believed that the cues were accurate, suggesting uncertainty may have less of an influence in our study. A further limitation of the current study was a relatively small sample size, which limited our ability to analyse potential gender differences in the effects observed.

## Conclusion

Prior threatening cues interfere with the effect of safety cues on pain perception, through a priming effect which enhances early cortical representation of sensory stimuli in the MCC, an area associated with adaptive control in aversive situations. Fearful individuals demonstrate an increased early neural response to threatening predictions, providing neurophysiological insights into the fear-avoidance model. The findings in this study provide important neurophysiological insights into how negative preconceptions may interfere with positive expectations about pain, resulting in a reduced therapeutic outcome. Moreover, the enhanced priming of early sensory mechanisms in fearful individuals identified in our study further strengthens the theory that pain-related fear may be an important target when managing pain. Together these results suggest an assessment of patient’s expectations and feelings towards pain and treatments, before delivering positive information about treatments, may be important in maximising therapeutic outcome. Further studies in patients are required to establish if this approach can provide a method of phenotyping patients’ different styles of cognitive and emotional appraisal of threatening stimuli.

## Supporting information

S1 Dataset Individual pain reports, fear of pain questionnaire scores and N2 laser evoked potentials are provided. (XLSX)
